# A Case of Successful Treatment with Unilateral Oophorectomy in a Patient with Resistant Polycystic Ovary Syndrome

**DOI:** 10.1155/2020/8893000

**Published:** 2020-11-25

**Authors:** S. Pathmanathan, I. Ranathunga, N. P. Somasundaram

**Affiliations:** ^1^Diabetes and Endocrinology Unit, District General Hospital-Kaluthara, Kaluthara, Sri Lanka; ^2^Diabetes and Endocrinology Unit, National Hospital of Sri Lanka, Colombo, Sri Lanka

## Abstract

**Background:**

Polycystic ovary syndrome (PCOS) is a common endocrine disorder with heterogeneous etiology. Typical features consist of oligo/anovulation, polycystic ovaries, and features of hyperandrogenism. Pathogenesis is multifactorial, and positive family history may have a predisposition for disease development. The syndrome is associated with multiple metabolic and nonmetabolic entities. As the disease is involved with multiple adverse outcomes, the successful treatment is pivotal. Among the more advanced options, the unilateral oophorectomy is considered as a last resort to alleviate the symptoms. *Case Presentation*. A 29-year-old female presented to us with oligomenorrhea, severe hirsutism, androgenic pattern hair loss, acne, increased skin pigmentation, and secondary subfertility. On examination, she was obese with a body mass index (BMI) of 29.6 kg/m^2^. She had evidence of acanthosis nigricans, androgenic pattern balding, acne, dorsal, supraclavicular fat deposition, and moderate-severe hirsutism. Investigations confirmed excess right ovarian testosterone secretion which led to the ultimate management with right oophorectomy with successful alleviation of clinical features.

**Conclusions:**

The multifaceted medical treatment comprises the first-line therapy in PCOS. Surgery is considered as a second-line option in resistant PCOS following failure of initial therapeutic options. We report a case of resistant polycystic ovary syndrome with secondary subfertility and moderate-to-severe hirsutism who was successfully treated with unilateral oophorectomy with favorable results.

## 1. Introduction

Polycystic ovary syndrome is a common endocrine disorder in premenopausal women. The prevalence of the disease varies depending on the population studied and ranges from 4–20% [1, 2]. The syndrome is characterized by the presence of oligo/anovulation, polycystic ovary appearance on imaging and clinical and biochemical features of hyperandrogenism. The diagnostic criteria had been evolved over past few decades, and many experts recommend the Rotterdam criteria in making the diagnosis at present [3]. The pathogenesis is thought to be multifactorial with possible involvement of genetic and environmental factors [4]. The disorder is associated with numerous other metabolic and nonmetabolic diseases which render to the increased morbidity and mortality linked to the disease [5–8]. Multiple treatment options are available for successful treatment of the disease which are mainly guided by the treatment goals [9]. Surgery has been used as a possible treatment option for infertility resistant to other treatment methods but is less popular as a method to treat hyperandrogenic symptoms resistant to other treatment [10].

## 2. Case Report

A 29-year-old previously healthy mother of one child presented with oligomenorrhea, severe hirsutism, androgenic pattern hair loss, acne, increased skin pigmentation, and secondary subfertility over five-year duration. Her cycles occurred once in every 3-4 months with bleeding lasting 5–7 days. She did not complain of associated dysmenorrhea. She has attained menarche at the age of 13 years and had regular menstrual cycles thereafter until five years back. A family history of a similar endocrinopathy was not evident.

**Figure 1:**
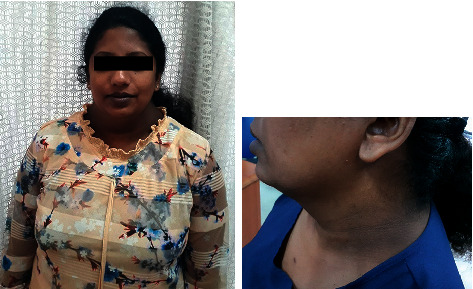
The index patient demonstrating central obesity and acanthosis nigricans.

On examination, she was obese with a body mass index (BMI) of 29.6 kg/m^2^. She had evidence of acanthosis nigricans, androgenic pattern balding, acne, and dorsal and supraclavicular fat deposition without any other specific features of Cushing's syndrome (Figure 1). Skin had a generalized increased in pigmentation without any predisposition to specific areas. Ferriman–Gallwey score was sixteen out of thirty-three which was suggestive of moderate-severe disease. Her systemic examination was nonsignificant.

On laboratory evaluation, her total testosterone was 215 ng/dL, luteinizing hormone (LH) was 7.27 IU/L, and follicle-stimulating hormone (FSH) was 7.42 IU/L. The thyroid profile, serum prolactin, dehydroepiandrosterone sulfate (DHEAS), and stimulated 17-hydroxyprogesterone (OHP) were found to be normal. Imaging with transvaginal ultrasound scan and MRI-pelvis revealed enlarged ovaries with multiple peripheral cysts which was suggestive of bilateral polycystic ovary syndrome. CECT of adrenal glands was found to be normal. A summary of the investigations is depicted in Table 1.

## 3. Conclusion: Features Suggestive of Polycystic Ovaries

**Figure 3:**
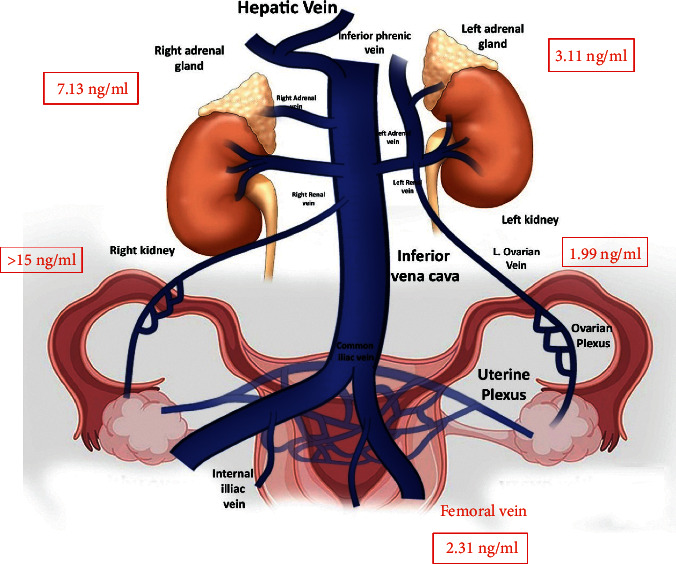
Bilateral ovarian/adrenal venous sampling: serum testosterone levels.

For further evaluation of markedly elevated serum total testosterone levels, she underwent bilateral adrenal and ovarian venous sampling which revealed a significant high testosterone levels being secreted from the right ovary (Figure 3, Table 2).

**Figure 4:**
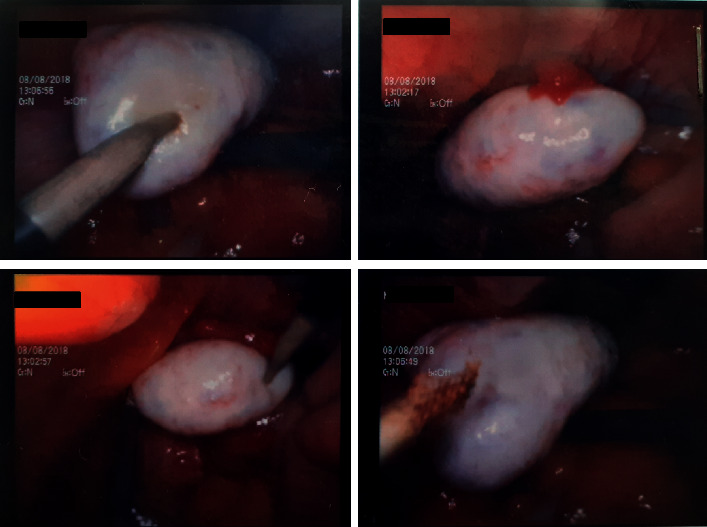
Enlarged bilateral ovaries with typical polycystic appearance.

She was started on medical nutrition therapy, metformin, antiandrogen (spironolactone), local therapy, and later a trial of GnRH analogs which were all found to be ineffective. To improve fertility, she underwent bilateral ovarian drilling without any successful outcomes. Enlarged bilateral ovaries with typical polycystic appearance were evident on laparoscopy (Figure 4).

**Figure 5:**
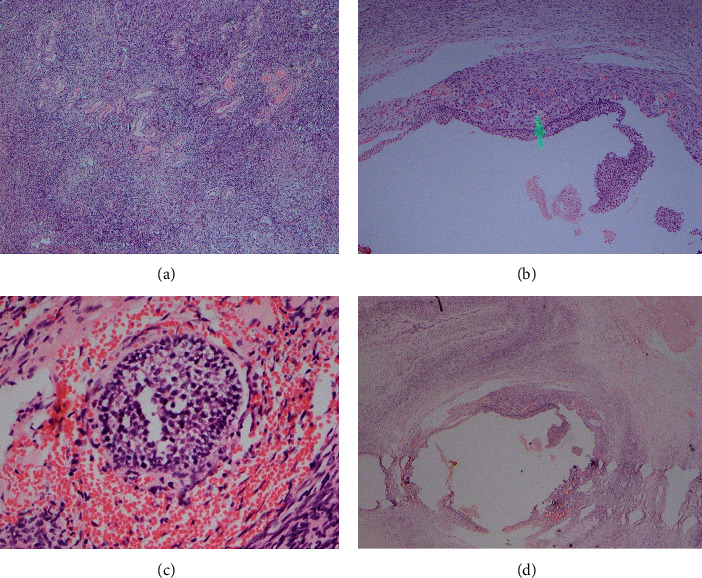
Right ovary histology: ovarian tissue comprises multiple ovarian follicles, some with luteinized cells. Areas of stromal hyperplasia are seen; however, luteinized cell collections are not seen. There is no evidence of hyperplasia or malignancy. Conclusion of histological features is consistent with polycystic ovary with stromal hyperplasia. (a) Stromal hyperplasia. (b) Ovarian follicle with luteinized cells. (c) Multiple follicles (400x). (d) Multiple follicles (40x).

Failing all the above treatments, she underwent right-sided oophorectomy which was the culprit ovary for the highest testosterone production as the last resort. Histology was compatible with polycystic ovary disease with multiple ovarian follicles, some with luteinized cells and stromal hyperplasia (Figure 5). In contrast, the presence of steroidogenically active nests of luteinized theca cells scattered in the ovarian stroma is seen in ovarian hyperthecosis. These cell clusters arise from the differentiation of the ovarian interstitial cells into luteinized stromal cells [11]. The clinical presentation of the two conditions may be similar though PCOS is commonly seen in premenopausal females, while ovarian hyperthecosis is more prevalent in the postmenopausal age group.

Following surgery, she had improvement of her clinical symptoms including hirsutism, regularization of the menstrual cycles, and normalization of total serum testosterone levels (46 ng/dL) and is currently under our follow-up for more than one year without any clinical or biochemical deterioration.

## 4. Discussion

PCOS is mainly a clinical diagnosis made in conjunction with recommended diagnostic criteria. Though multiple diagnostic criteria are available at present, the commonly used is the criteria defined by the European Society for Human Reproduction and Embryology/American Society for Reproductive Medicine (ESHRE/ASRM), Rotterdam criteria [3]. Nevertheless, other supportive investigation including biochemistry, imaging, and histology contribute to the diagnostic accuracy. Selective venous catheterization, which is an invasive modality of investigation, has been successfully employed to aid in the localization and the diagnosis of the source of androgen secretion. Even though ovarian venous sampling is not used as a routine investigation, the utilization of the test can rule out possible malignant causes and plan more selective, third-line treatment options in the presence of significant hyperandrogenism as in the index patient [12]. Nonetheless, difficulty in catheterization and the invasiveness of the procedure may limit its successful use [13].

The treatment of the disease should be guided by the goals of therapy [9]. First-line treatment consists of lifestyle interventions including diet and exercise targeted in achieving significant weight loss [9]. Further treatment will depend on the fertility wishes which include hormonal contraceptives, metformin therapy, clomiphene citrate, letrozole, and gonadotrophin therapy [9]. Surgery has been rarely practiced as a possible treatment option for infertility resistant to other treatment methods [14]. Out of the available surgical options, laparoscopic ovarian drilling is an established treatment option for infertility for women resistant to medical therapy [15]. Though the exact mechanism is not clear, the destruction of ovarian follicles and stroma leading to a reduction in androgen and inhibin levels, which drives the pathogenesis and a subsequent rise in follicle-stimulating hormone (FSH) levels, is the most possible explanation [16]. Utilization of surgical options such as ovarian drilling, wedge resection, unilateral or bilateral oophorectomy is less considered for the management of resistant hyperandrogenism manifested by hirsutism, acne etc., though some studies have demonstrated improved clinical outcomes [10, 17, 18]. Literature suggests the possibility of decreased androgen and LH levels following surgery which may contribute the favorable clinical outcomes, though the results are inconsistent [10]. Unilateral oophorectomy remains an uncommon option that can be exerted in resistant patients who has undergone multiple medical and conservative surgical treatment. However, theoretically, removal of one ovary can lead to less production of androgens and can lead to improvement of clinical features and fertility [14]. Studies have demonstrated that unilateral oophorectomy can restore regular menstruation and fertility in majority [14]. Ovarian surgery has not generally been demonstrated to have an effect on the severity of hirsutism, but has been suggested to decrease hair growth rate in some women [19, 20]. There are insufficient and conflicting data to assess acne as an outcome [18, 21]. The possibility of the disease recurrence despite initial improvement at a later date remains a possibility, and active vigilance should be carried out indefinitely [14]. The index patient had a significant improvement in the androgenic features including hirsutism, acne, and androgenic pattern hair loss as well as regularization of menstrual cycles following surgery.

This case highlights the importance of further evaluation of resistant PCOS patients with more advanced investigations such as ovarian venous sampling and consideration of aggressive surgical interventions such as unilateral oophorectomy which can yield successful outcomes. Furthermore, consideration of unilateral oophorectomy in the event of unilateral testosterone hypersecretion can be limited in the reproductive age group due to concomitant fertility wishes. Nevertheless, further study of similar case reports and more detailed research will be beneficial in the future and will add up to the options available for treatment in the resistant patients for conventional treatment.

## 5. Conclusions

In conclusion, here, we have described a rare case of resistant polycystic ovary syndrome who presented to us with severe hirsutism and was found to have unilateral secretion of very high testosterone levels. She was successfully treated with unilateral oophorectomy of the culprit ovary indicating that surgery can be considered in resistant patients as a last resort.

## Figures and Tables

**Figure 2:**
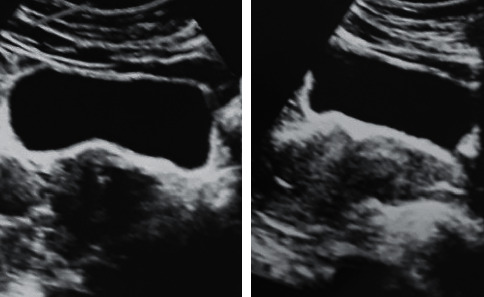
Both ovaries are enlarged; R/ovary: 14 ml; L/ovary: 17 ml; multiple small follicles are seen at the periphery of the ovary; prominent stroma seen.

**Table 1:** Summary of investigations.

Investigation	Result	Reference range
Serum total testosterone	215 ng/dL	14–76 ng/dL

LH	7.27 IU/L	1.8–11.78 IU/L (follicular phase)

FSH	7.42 IU/L	3.03–8.08 IU/L (follicular phase)

17-Hydroxyprogesterone (17-OHP)	3.954 nmol/L	<2.1 nmol/L

Synacthen stimulated 17-OHP	4.915 nmol/L	<9.6 nmol/L

Dehydroepiandrosterone sulfate (DHEAS)	23.1 *µ*g/dL	35–430 *µ*g/dL

LDDST suppressed serum total testosterone	118.67 nmol/L	<50%

Low-dose dexamethasone suppression test (LDDST)	13.79 nmol/L	<50 nmol/L

Fasting blood glucose	86 mg/dL	<100 mg/dL

Oral glucose tolerance test (2 hour)	128 mg/dL	<140 mg/dL

Lipid profile	Cholesterol: 248 mg/dL HDL: 39 mg/dL LDL: 183 mg/dL TG: 132 mg/dL	<200 mg/dL >60 mg/dL < 130 mg/dL < 150 mg/dL

TSH/fT4	1.18 *µ*IU/L/1.42 ng/dL	0.55–4.78 *µ*IU/L/0.89–1.76 ng/dL

Postoperative serum total testosterone	46 ng/dL	14–76 ng/dL

Transvaginal scan	Both ovaries are enlarged, R/ovary: 14 ml, L/ovary: 17 ml, multiple small follicles are seen at the periphery of the ovary, prominent stroma is seen Features suggestive of polycystic ovaries (Figure 2)	

MRI-pelvis	Both ovaries are enlarged, R/ovary: 16 ml, L/ovary: 20 ml, multiple tiny follicles (>20) in both ovaries with a peripheral distribution. The size of the follicles is less than 10 mm. Prominent stroma is noted.	

CECT-adrenal glands	Normal	

**Table 2:** Bilateral ovarian/adrenal venous sampling.

	Left ovary	Right ovary	Left adrenal	Right adrenal	Femoral vein
Serum total testosterone	1.99 ng/ml	>15 ng/ml	3.11 ng/ml	7.13 ng/ml	2.31 ng/ml
Serum cortisol			3820 nmol/L	303.5 nmol/L	348 nmol/L

## Data Availability

Clinical details and results of investigations are documented in bed head tickets. Bed head tickets are available in the record room of District General Hospital, Kaluthara, and National Hospital of Sri Lanka. Most of the original reports are with the patient.

## Abbreviations

PCOS: Polycystic ovary syndrome
BMI: Body mass index
LH: Luteinizing hormone
FSH: Follicle-stimulating hormone
DHEAS: Dehydroepiandrosterone sulfate
17-OHP: 17-hydroxyprogesterone
MRI: Magnetic resonance imaging
CECT: Contrast-enhanced computed tomography
LDDST: Low-dose dexamethasone suppression test
THS: Thyroid stimulating hormone
ESHRE/ASRM: European Society for Human Reproduction and Embryology/American Society for Reproductive Medicine.

## References

[1] Azziz R., Woods K. S., Reyna R., Key T. J., Knochenhauer E. S., Yildiz B. O. (2004). The prevalence and features of the polycystic ovary syndrome in an unselected population. *The Journal of Clinical Endocrinology & Metabolism*.

[2] Asuncion M., Calvo R. M., San Millan J. L., Sancho J., Avila S., Escobar-Morreale H. F. (2000). A prospective study of the prevalence of the polycystic ovary syndrome in unselected Caucasian women from Spain. *Journal of Clinical Endocrinology & Metabolism*.

[3] Teede H. J., Misso M. L., Costello M. F (2018). Recommendations from the international evidence-based guideline for the assessment and management of polycystic ovary syndrome. *Fertility and Sterility*.

[4] Rosenfield R. L., Ehrmann D. A. (2016). The pathogenesis of polycystic ovary syndrome (PCOS): the hypothesis of PCOS as functional ovarian hyperandrogenism revisited. *Endocrine Reviews*.

[5] Dunaif A., Segal K. R., Futterweit W., Dobrjansky A. (1989). Profound peripheral insulin resistance, independent of obesity, in polycystic ovary syndrome. *Diabetes*.

[6] Setji T. L., Holland N. D., Sanders L. L., Pereira K. C., Diehl A. M., Brown A. J. (2006). Nonalcoholic steatohepatitis and nonalcoholic Fatty liver disease in young women with polycystic ovary syndrome. *The Journal of Clinical Endocrinology & Metabolism*.

[7] Ford E. S., Giles W. H., Mokdad A. H. (2004). Increasing prevalence of the metabolic syndrome among U.S. Adults. *Diabetes Care*.

[8] Deeks A. A., Gibson-Helm M. E., Teede H. J. (2010). Anxiety and depression in polycystic ovary syndrome: a comprehensive investigation. *Fertility and Sterility*.

[9] Legro R. S., Arslanian S. A., Ehrmann D. A. (2013). Diagnosis and treatment of polycystic ovary syndrome: an Endocrine Society clinical practice guideline. *The Journal of Clinical Endocrinology & Metabolism*.

[10] Johnson N., Wang K. (2003). Is ovarian surgery effective for androgenic symptoms of polycystic ovarian syndrome?. *Journal of Obstetrics and Gynaecology*.

[11] Judith N. B. (2004). Nonneoplastic disorders. *Pathology of the Ovary*.

[12] Kaltsas G. A., Mukherjee J. J., Kola B. (2003). Is ovarian and adrenal venous catheterization and sampling helpful in the investigation of hyperandrogenic women?. *Clinical Endocrinology*.

[13] Wentz A. C., White R. I., Migeon C. J., Hsu T. H., Barnes H. V., Jones G. S. (1976). Differential ovarian and adrenal vein catheterization. *American Journal of Obstetrics and Gynecology*.

[14] Kaaijk E. M. (1999). Clinical outcome after unilateral oophorectomy in patients with polycystic ovary syndrome. *Human Reproduction*.

[15] Mitra S., Nayak P. K., Agrawal S. (2015). Laparoscopic ovarian drilling: an alternative but not the ultimate in the management of polycystic ovary syndrome. *Journal of Natural Science, Biology and Medicine*.

[16] Flyckt R., Goldberg J. (2011). Laparoscopic ovarian drilling for clomiphene-resistant polycystic ovary syndrome. *Seminars in Reproductive Medicine*.

[17] Naether O. G. J., Baukloh V., Fischer R., Kowalczyk T. (1994). Surgery: long-term follow-up in 206 infertility patients with polycystic ovarian syndrome after laparoscopic electrocautery of the ovarian surface. *Human Reproduction*.

[18] Amer S. A. K., Gopalan V., Li T. C., Ledger W. L., Cooke I. D. (2002). Long term follow-up of patients with polycystic ovarian syndrome after laparoscopic ovarian drilling: clinical outcome. *Human Reproduction*.

[19] Vejlsted H., Albrechtsen R. (1976). Biochemical and clinical effect of ovarian wedge resection in the polycystic ovary syndrome. *Obstetrics and Gynecology*.

[20] Hamerlynck J. (1982). Polycystic ovaries disease: one ovary too many?. *Lancet (London, England)*.

[21] Gjönnaess H. (1984). Polycystic ovarian syndrome treated by ovarian electrocautery through the laparoscope. *Fertility and Sterility*.

